# Impact of Palonosetron on Cough Suppression in Females Undergoing Sevoflurane-Remifentanil Anesthesia for Laparoscopic Cholecystectomy: A Randomized Trial

**DOI:** 10.3390/jpm11090887

**Published:** 2021-09-05

**Authors:** Ho-Young Gil, Ha-Yeon Kim, Hye-Sun Lee, Na-Young Kim, Ji-Eun Kim

**Affiliations:** 1 Department of Anesthesiology and Pain Medicine, Ajou University School of Medicine, 164, World Cup-ro, Yeongtong-gu, Suwon 16499, Korea; kilhoyoung@naver.com (H.-Y.G.); hayeon@aumc.ac.kr (H.-Y.K.); jhkyanb@gmail.com (N.-Y.K.); 2 Biostatistics Collaboration Unit, Yonsei University College of Medicine, Seoul 03722, Korea; HSLEE1@yuhs.ac

**Keywords:** emergence cough, female, palonosetron, remifentanil

## Abstract

Remifentanil has been used to suppress peri-extubation cough. Palonosetron, a 5-HT_3_ receptor antagonist, is an effective antiemetic, and 5-HT receptors mediate the cough reflex. We assessed the impact of palonosetron on effect-site concentration (Ce) of remifentanil for preventing emergence cough in females. Forty-five female patients undergoing laparoscopic cholecystectomy randomly received 0.075 mg of palonosetron (*n* = 21) or normal saline (*n* = 24) intravenously at the end of surgery. The remifentanil Ce for 50% (EC_50_) and for 95% (EC_95_) of patients were estimated via Dixon’s up-and-down method or isotonic regression. Using Dixon’s method, EC_50_ in the control group (1.33 ± 0.38 ng/mL) was comparable to that of the palonosetron group (1.42 ± 0.75 ng/mL) (*p* = 0.813). Using isotonic regression, EC_50_ (83% CIs) and EC_95_ (95% CIs) did not reveal significant differences between the control and the palonosetron groups (1.17 (0.86–1.43) and 1.90 (1.45–1.96) ng/mL and 0.88 (0.78–1.23) and 2.43 (1.94–2.47) ng/mL, respectively). No difference was found in the remifentanil Ce to suppress emergence cough in the palonosetron group compared with the control group. It may indicate no effect of palonosetron on antitussive activity of remifentanil.

## 1. Introduction

Cough during emergence from general anesthesia frequently occurs in intubated patients, with an incidence as high as 76% [1]. Tracheal stimuli from the endotracheal tube are perceived by the central and peripheral nervous system, producing the cough [2]. Cough serves a protective role in facilitating the clearance of an inhalation agent, secretions, and irritants. However, the peri-extubation cough may adversely affect patients undergoing surgery, due to complications, such as hematoma of surgical site, wound dehiscence, and increased intracerebral and intraocular pressures [3,4]. Therefore, various interventions have been tried to minimize cough for a smooth emergence [5,6,7].

Remifentanil is a potent ultrashort-acting opioid accompanied by rapid onset and offset of effect. The target-controlled infusion (TCI) of remifentanil enables smooth extubation alongside lowered complications [8]. In a meta-analysis of 70 studies, remifentanil was the most effective in decreasing the severe peri-extubation cough compared with fentanyl, dexmedetomidine, and lidocaine [9]. Further, it attenuated the increase in hemodynamic parameters without extending the extubation time [9].

Postoperative nausea and vomiting (PONV) is a common adverse effect of anesthesia and surgery. PONV is associated with patient dissatisfaction, prolonged hospital stay, and higher costs of care in addition to severe complications [10]. Palonosetron is a popular antiemetic used for PONV prevention with long-lasting efficacy due to greater binding affinity to the 5-hydroxytryptamine-3 (5-HT_3_) receptor [11]. In addition, palonosetron was more effective than ramosetron in preventing PONV during laparoscopic cholecystectomy [12].

The 5-HT receptors are present in the central and peripheral nervous system [13]. The antitussive property of opioids is mediated via activation of these receptors, which inhibits the cough [14,15]. Further, 5-HT receptor agonists play a role in antitussive therapy [16,17]. Therefore, we hypothesized that prior treatment with palonosetron increases the need for remifentanil in suppressing emergence cough. The study assessed the impact of palonosetron on the optimal effect-site concentration (Ce) of remifentanil to suppress emergence cough in females who underwent laparoscopic cholecystectomy.

## 2. Materials and Methods

### 2.1. Patients

This study was approved by the Ajou University Hospital Institutional Review Board (AJIRB-MED-THE-20-174, 9 July 2020) and registered at http://ClinicalTrials.gov (NCT04563260, 24 September 2020). Following adequate information about this study, all patients provided written informed consent. Female patients with ASA physical status of I, II, or III aged between 19 and 85 years who underwent elective laparoscopic cholecystectomy were included from October 2020 to March 2021. Exclusion criteria were: body mass index ≥30 kg/m^2^, uncontrolled hypertension, coronary disease, arrhythmia, acute and respiratory disease (asthma, chronic obstructive pulmonary disease, and upper airway infection within the last 2 weeks), prior use of an antitussive drug, and anticipated difficult airway.

### 2.2. Anesthesia 

None of the patients was premedicated. In the operating room, patients received standard monitoring including pulse oximetry for oxygen saturation, electrocardiography, noninvasive blood pressure measurement, and bispectral index (BIS). A balanced anesthesia was implemented with sevoflurane and remifentanil (Ultian, Hanlim Pharm. Co., Ltd., Seoul, Korea). The TCI of remifentanil was set using a commercial TCI pump (Orchestra Base Primea, Fresinus Vial, Sevres, France) and was based on Minto’s pharmacokinetics. Following pre-oxygenation, anesthesia was induced with propofol 2 mg/kg and remifentanil 3–5 ng/mL as Ce. After loss of consciousness, manual ventilation with sevoflurane and 100% oxygen was initiated, followed by rocuronium 1.0 mg/kg [18]. After confirming muscle relaxation with train-of-four counts of 0, orotracheal intubation was conducted with a cuffed endotracheal tube (inner diameter, 7.0 mm) using a videolaryngoscope. Cuff pressure was controlled to 20–25 mmHg by a hand pressure gauge. Ventilation was mechanically initiated with a tidal volume of 6–8 mL/kg and a 50% oxygen plus air, while maintaining EtCO_2_ at 35–40 mmHg. Anesthesia was continued using sevoflurane of 1 minimal alveolar concentration and remifentanil of ≤5 ng/mL as Ce to maintain a BIS level of hypnosis of 40–60 and mean arterial pressure (MAP) and heart rate (HR) values within 20% of the baseline values.

IV palonosetron 0.075 mg was administered to the experimental group 10 min prior to the end of surgery, whereas the control group received the same volume of IV normal saline according to group assignments. IV acetaminophen 100 mg was administered to both groups for postoperative analgesia. Simultaneously, the remifentanil Ce was set at the predetermined Ce. IV sugammadex 2 mg/kg was injected at the end of surgery to reverse the neuromuscular block. The secretions in the tracheal tube or the pharynx were suctioned. After confirming the train-of-four ratio ≥0.9, sevoflurane was discontinued, and the fresh gas flow was increased to 5 L/min. Manual ventilation was started to maintain an EtCO_2_ of 40–45 mmHg in accordance with the patient’s spontaneous breath, followed by a verbal command to open the eyes without any stimulus. After eye opening upon verbal command and confirming adequate spontaneous ventilation, the tracheal tube cuff was deflated with a syringe, and the endotracheal tube was removed along its curve in the climax of the inspiration. Immediately, remifentanil was discontinued, and 100% oxygen was supplied via a facial mask. If there was secretion in the mouth and pharynx, it was suctioned again. The patients were then transferred to the post-anesthesia care unit (PACU).

### 2.3. Definition and Study Protocol

Emergence cough was assessed after discontinuing sevoflurane until 3 min after extubation. The cough level was classified as: 0 = no cough, 1 = single (no sudden contraction of abdominal muscle), 2 = more than one episode of nonsustained cough, and 3 = sustained and repetitive cough (H.Y.K. and N.Y.K.). Because level 1 had no clinical meaning, we deemed it as success. Two investigators were informed of the predetermined remifentanil Ce but blinded to the group assignment. Extubation time was defined as the duration from sevoflurane cessation until endotracheal extubation.

Patients were randomly assigned to either the control group or the palonosetron group using a computer-generated random table (http://www.random.org, 10 July 2020) and the concealed envelopes method (J.E.K.). They were sequentially enrolled until completing at least 6 pairs of failure-success and 20 patients according to Dixon’s up-and-down method. If one group reached 6 pairs of failure-success, then all the following patients were enrolled in the other group [19,20]. Dixon’s sequential allocation design was adopted to determine the remifentanil Ce in each group. The first patient was started with 2.0 ng/mL. The predetermined Ce of the next patient was determined by the cough response during emergence of the prior patient. If the patient did not cough or coughed at level 1 during emergence, it was defined as successful smooth emergence. The predetermined Ce of the next patient was decreased by a concentration of 0.5 ng/mL compared with the prior patient. If the patient coughed at levels 2 and 3, we defined them as fail, and the predetermined Ce of the next patient was increased by a concentration of 0.5 ng/mL.

The number of intubation attempts was recorded, and the HR and MAP were recorded at 4 time-points: baseline (before induction), the end of surgery, after extubation, and at PACU. The end-tidal concentration of sevoflurane was recorded when opening the eyes on verbal command without stimuli. Respiratory complications such as hypoventilation (respiratory rate < 8 breaths/min), laryngospasm, and desaturation (oxygen saturation < 95%) were recorded. Immediately on arrival in the PACU, the sedation score using the Ramsay Sedation Scale (six levels: 1 = anxious and agitated or restless or both; 6 = no response) was assessed by the attending nurse. Nausea (1 = none, 2 = mild, 3 = moderate, and 4 = severe), vomiting, shivering, headache, and pain score using an 11-point numerical rating scale (NRS: 0 = no pain, 10 = worst pain) were also assessed. IV dexamethasone 5 mg was administered for nausea ≥3, and a fentanyl dose of 0.5 mcg/kg was administered in the event of NRS ≥ 5. After 20 min of assessment, pain and nausea were re-evaluated. The patients were transferred to a ward when they reached a total modified Aldrete score of 8, including respiratory score of 2. 

### 2.4. Statistical Analysis

The primary outcome was to estimate the EC_50_ and EC_95_ of remifentanil to suppress emergence cough depending on whether or not palonosetron was used. The EC_50_ was calculated as a mean value of remifentanil Ce and compared using the independent *t*-test. EC_50_ and EC_95_ were also calculated using the isotonic regression method in the basis of a pooled-adjacent-violators algorithm (PAVA), and 83% and 95% confidence intervals (CIs) were calculated through a boot strap method [19]. When EC_50_ and EC_95_ did not overlap at the 83% CIs and 95% CIs, they were considered as statistically significant differences. For sample size, patient enrollment was stopped in each group when completing at least 6 pairs of failure-success and 20 patients according to Dixon’s up-and-down method [19].

Values were expressed as the mean ± standard deviation (SD), the median (interquartile range), or the number of patients. Normality was determined using the Shapiro–Wilk test. Continuous variables were analyzed using Student’s *t*-test or the Mann–Whitney test, depending on normality. Categorical variables were compared using the Chi-square test or Fisher’s exact test. Repeated-measured variables were analyzed using a linear mixed model. A *p* value of <0.05 was considered statistically significant. SAS (version 9.4, SAS Inc., Cary, NC, USA) and R package, version 4.0.5 (http://www.R-project.org, 25 April 2021) was used for statistical analyses.

## 3. Results

### 3.1. Study Population 

**Figure 1:**
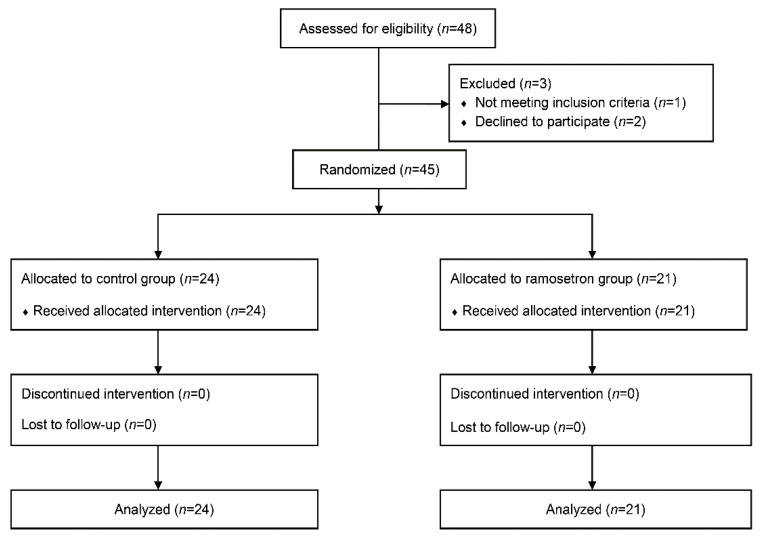
The CONSORT flow diagram of patients.

Three of the 48 enrolled patients refused to participate. Ultimately, 45 patients were randomized and included in the final analysis (Figure 1).

### 3.2. Baseline Characteristics

**Figure 2:**
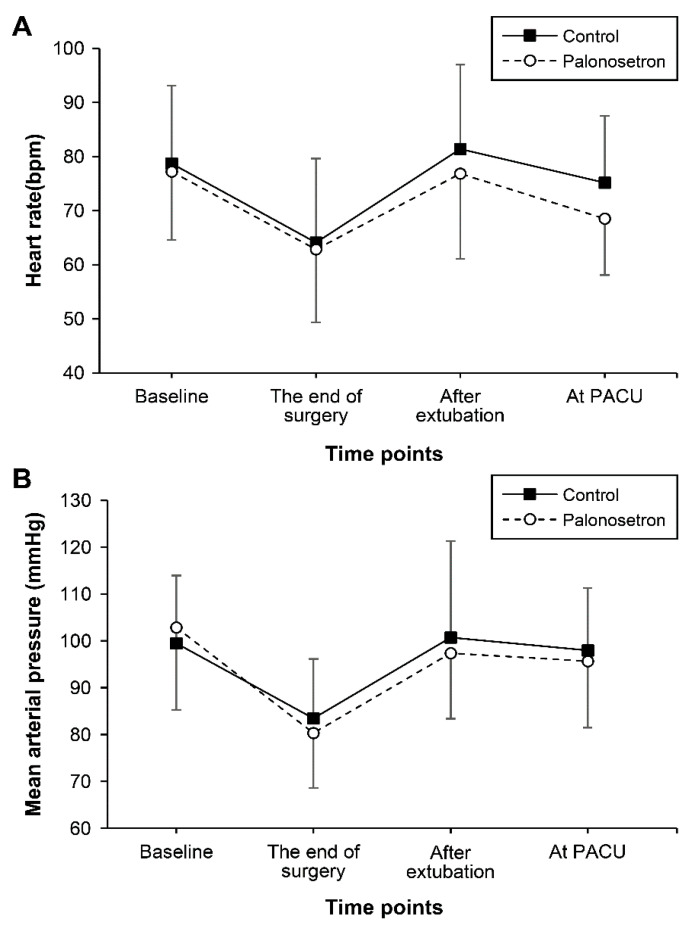
The (**A**) mean arterial pressure and (**B**) heart rate during the perioperative period. Data are expressed as the mean ± standard deviation. PACU, post-anesthesia care unit. “Baseline” means before induction.

Demographic and intraoperative characteristics of patients did not differ between the two groups (Table 1). The MAP and HR were also comparable throughout perioperative period (Figure 2).

### 3.3. Optimal Ce of Remifentanil

**Figure 3:**
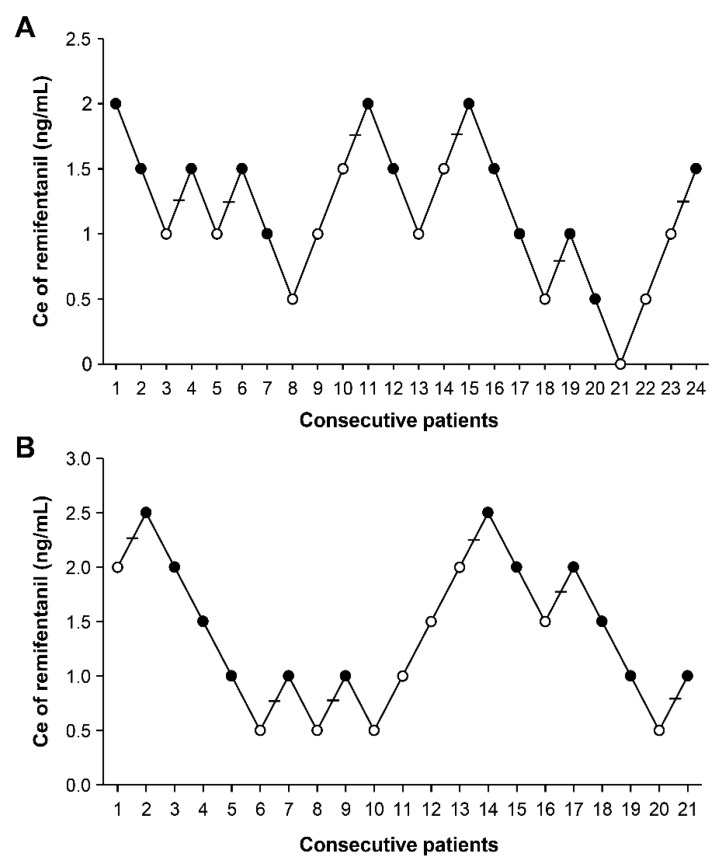
Sequences of effect-site concentration (Ce) of remifentanil to prevent emergence cough during extubation by Dixon’s up-and-down methods. Horizontal bars represent crossover midpoints (i.e., failure to success). The mean EC_50_ of remifentanil Ce for suppressing emergence cough was calculated from cross-over pairs of success (closed circle) and failure (open circle) in (**A**) 24 patients in the control group and (**B**) 21 patients in the palonosetron group.

**Figure 4:**
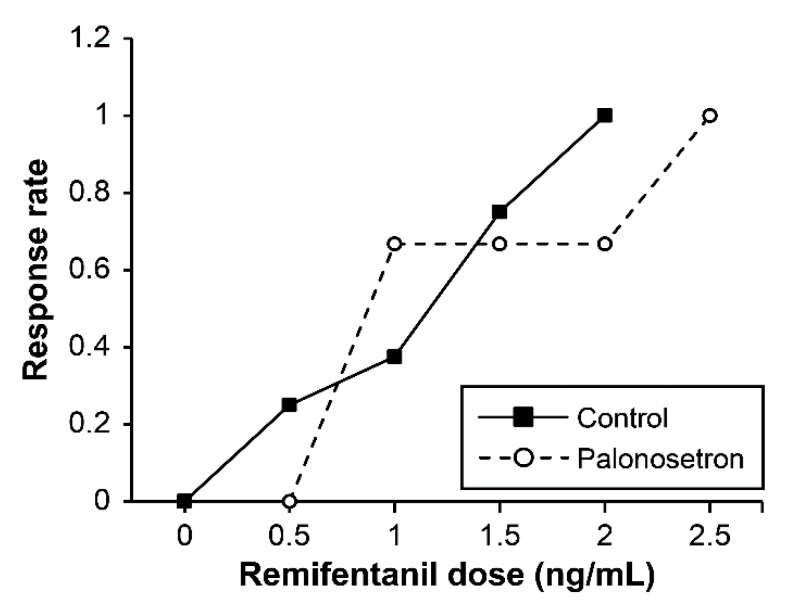
Pooled-adjacent-violators algorithm (PAVA) response rates in control (closed square) and palonosetron (open circle) groups of patients. The PAVA response rate means the ratio of the number of successful patients to the number of total patients at each remifentanil Ce in each group.

The sequence of failures and successes on smooth emergence based on Dixon’s method is showed in Figure 3, and an isotonic regression curve in the basis of PAVA response rate is presented in Figure 4. EC_50_ and EC_95_ values of remifentanil Ce for preventing emergence cough estimated with Dixon’s method or isotonic regression are described in Table 2. Based on Dixon’s method, EC_50_ of remifentanil Ce was 1.33 ± 0.38 ng/mL in the control group, which was comparable to 1.42 ± 0.75 ng/mL in the palonosetron group (*p* = 0.813). Based on isotonic regression, EC_50_ of the remifentanil Ce was 1.17 (83% CI, 0.86–1.43) ng/mL in the control group and 0.88 (0.78–1.23) ng/mL in the palonosetron group. EC_95_ of the remifentanil Ce was 1.90 (95% CI, 1.45–1.96) ng/mL in the control group and 2.43 (1.94–2.47) ng/mL in the palonosetron group. EC_50_ and EC_95_ values did not overlap, suggesting no significant difference in optimal remifentanil Ce between the two groups.

### 3.4. Emergence and Recovery Data

The emergence and recovery data are presented in Table 3. During emergence from anesthesia, bradypnea was observed in three patients (remifentanil Ce: 0.5, 1.0, and 1.5 ng/mL) in the control group and in one patient (remifentanil Ce, 1.0 ng/mL) in the palonosetron group. However, these patients were restored to a normal respiratory pattern immediately via deep breathing using a facial mask. The sedation status of six levels showed significant difference between the two groups (*p* = 0.028) in the PACU; however, most patients showed sedation status within the range of awake levels (sedation levels: 1, 2, and 3) except for one patient in the control group (*p* > 0.999). In addition, no shivering occurred; however, three patients complained of a headache (two in the control group and one in the palonosetron group).

## 4. Discussion

In this study, a balanced anesthesia using sevoflurane and remifentanil TCI was implemented in female patients to evaluate the impact of palonosetron on the optimal remifentanil Ce for smooth emergence. As a result, the EC_50_ and EC_95_ values of remifentanil for suppressing emergence cough at extubation were not significantly different between the two groups. Further, the emergence and recovery data were comparable.

Several possibilities may explain the lack of increase in remifentanil requirement for smooth emergence in the present study. First, the opioid system is known to interact functionally with the serotonergic system in the central nervous system. Multiple opioid receptors differentially modulate 5-HT efflux in the brain [14]. These opioid receptors mediate the hypotensive response induced by central 5-HT_3_ receptor stimulation [21]. 5-HT_1_ receptors modulate opioid release in the spinal cord, and its agonists prevent opioid-induced respiratory depression, analgesia, and sedation [22,23]. In addition, 5-HT_3_ receptor antagonists such as ondansetron reduce opioid withdrawal behaviors [24]. Likewise, 5-HT_3_ receptors mediate the vomiting reflex, processing of pain, cognition, and anxiety control [25]. However, few studies elucidate the role of 5-HT_3_ receptors in the antitussive effect of opioids. Although tussigenic and antitussive effects of palonosetron on opioids in the central nervous system were not delineated by our study, it might not have a definitive impact on the results, because the human 5-HT_3_ receptor is a target relatively less sensitive to remifentanil than to morphine [26].

Second, 5-HT_3_ receptors exist in the respiratory tract. Airways receive a dense supply of sensory nerve fibers that originate mainly in the jugular and nodose vagal ganglia embryologically [27]. Although jugular ganglia do not express 5-HT_3_ receptors, nodose ganglia expressing 5-HT_3_ receptors are strongly activated by 5-HT or a 5-HT_3_ agonist [13]. Therefore, the injection of 5-HT or a 5-HT_3_ agonist inhibits the cough response triggered by mechanical stimulation of trachea in animal studies [13,28]. In addition, 5-HT has been shown to inhibit the cough reflex in humans at the peripheral site because 5-HT does not cross the blood-brain barrier [29]. Therefore, 5-HT receptor agonists were proposed as one of the novel antitussive treatments [16,17]. In this connection, we hypothesized that palonosetron, as a 5-HT_3_ receptor antagonist, induces cough and increases the remifentanil Ce. However, there was no increase of remifentanil Ce in the palonosetron group, presumably due to the critical role of jugular, rather than nodose, vagal ganglia in the induction and sensitization of cough [27].

Third, 5-HT_3_ receptors are known to be expressed in the central and peripheral nervous system and integrate the processing of pain [25,30]. 5-HT activates presynaptic 5-HT_3_ receptors on spinal afferents, which transmit nociceptive input from the periphery to the brain, thereby increasing pain and reflex responses [31]. By contrast, the 5-HT_3_ receptor antagonist (ondansetron) prevents the development of chronic pain in rats [32]. As a nociceptive mechanism, the role of 5-HT_3_ was established especially in formalin-induced nociception in mice [33]. The 5-HT_3_ receptor is involved in the release of pain mediators, such as substance P in the nerve, and a 5-HT_3_ receptor antagonist affects substance P-mediated hyperalgesia [30]. In humans, treatment with 5-HT_3_ receptor antagonists alleviate chronic pain such as neuropathic pain, rheumatoid disease, migraine, and fibromyalgia [30,34]. In addition, granisetron, a specific 5-HT_3_ receptor antagonist, increases the pain threshold in healthy males, indicating its potential local anesthetic role as an alternative to lidocaine [35]. Further, palonosetron pretreatment alleviates the pain induced by propofol as well as rocuronium injections, thereby reducing withdrawal movement during general anesthesia [36,37]. In view of the pain associated with endotracheal intubation, the antinociceptive effects of palonosetron during extubation in our study might neutralize its cough-inducing effect mentioned above.

In the present study involving females aged between 19 and 85 years and using acetaminophen for postoperative analgesia, EC_50_ of remifentanil Ce was 1.42 ± 0.75 ng/mL after palonosetron administration compared with 1.33 ± 0.38 ng/mL in the control group. However, in two previous studies conducted at the same institute, elderly females received ketorolac for postoperative analgesia, and EC_50_ values of remifentanil Ce after ramosetron administration were higher (1.56 ± 0.26 ng/mL and 2.08 ± 0.47 ng/mL) [38,39]. This comparison may be reasonable because, except for these two variables (age and ketorolac), other conditions remained constant, including the inhalation agent (sevoflurane), endotracheal tube (cuffed, internal diameter of 7.0 mm), sex (female), neuromuscular reversal drug (sugammadex), and surgical type (elective laparoscopic cholecystectomy). Further, the higher Ce of ramosetron may be underestimated because perioperative nonsteroidal anti-inflammatory drugs (NSAIDs) such as ketorolac are more effective than acetaminophen in reducing the risk of PONV [40]. Possibly, ramosetron, the newer second-generation 5-HT_3_ receptor antagonist, may be able to significantly increase remifentanil Ce for smooth extubation in contrast to palonosetron. Further studies are needed to shed additional light on these findings.

There are several limitations. First, adults and elderly females were included in the present study. However, age was considered because of the possible age-related differences in the cough reflex or the pharmacokinetics of opioid [41,42]. Second, cough severity might be a subjective interpretation on the patient’s actions, thus posing the issue of interobserver variability and bias. Third, palonosetron was injected at the end of surgery, not at anesthetic induction. However, the injection timing of palonosetron is not yet settled, unlike that of dexamethasone or ramosetron [40]. Fourth, the incidence of PONV did not differ between the groups despite an ethical concern of no preventive treatment of PONV in the control group. Prophylactic IV acetaminophen known to reduce PONV might affect this nondifference [40]. Fifth, the postoperative pain score was high even for laparoscopic surgery. The use of long-acting opioids may be proper for laparoscopic cholecystectomy in clinical practice. Sixth, an experimental animal study may be needed to establish the role of 5-HT_3_ receptor antagonists in the antitussive effects of opioid. Finally, the EC_50_ values showed different tendencies depending on Dixon’s or isotonic regression methods, although there were no statistical differences. Further study with a large sample size may be needed to overcome a potential lack of power.

## 5. Conclusions

There was no difference in the optimal remifentanil Ce to suppress emergence cough between the palonosetron and the control groups. It may indicate no effect of palonosetron on antitussive activity of remifentanil. The remifentanil Ce to suppress emergence cough may not be adjusted in clinical practice when used in female patients previously treated with palonosetron.

## Figures and Tables

**Table 1:** Demographic and intraoperative characteristics of patients.

	Control (*n* = 24)	Palonosetron (*n* = 21)	*p*-Value
Age (years)	48.0 ± 13.2	52.7 ± 12.2	0.222
Height (cm)	159.3 ± 6.1	157.4 ± 5.4	0.282
Weight (kg)	63.8 ± 10.2	59.1 ± 9.7	0.126
ASA classification 1/2/3	15/9/0	12/8/1	0.871
Intubation attempts once/twice	24/0	21/0	0.489
Operation time (min)	45 (35–50)	40 (35–50)	0.756
Anesthesia time (min)	75 (65–80)	75 (65–80)	0.881

Values are expressed as the mean ± standard deviation, median (interquartile range), or number. ASA, American Society of Anesthesiologists.

**Table 2:** Optimal Ce of remifentanil for suppressing emergence cough.

	Control (*n* = 24)	Palonosetron (*n* = 21)	*p*-Value
Dixon’s method			
EC_50_ of remifentanil Ce (ng/mL)	1.33 ± 0.38	1.42 ± 0.75	0.813
Isotonic regression method			
EC_50_ of remifentanil Ce (ng/mL)	1.17 (0.86–1.43)	0.88 (0.78–1.23)	
EC_95_ of remifentanil Ce (ng/mL)	1.90 (1.45–1.96)	2.43 (1.94–2.47)	

EC_50_ expressed as the mean ± standard deviation were determined by Dixon’s method, and EC_50_ (83% CI) and EC_95_ (95% CI) were determined by the isotonic regression method. Ce, effect-site concentration; CI, confidence interval.

**Table 3:** Emergence and recovery data.

	Control (*n* = 24)	Palonosetron (*n* = 21)	*p*-Value
During emergence			
Extubation time (min)	11.0 ± 3.2	11.1 ± 3.5	0.942
EtSevo at eye opening (%)	0.25 ± 0.1	0.21 ± 0.09	0.155
Respiratory complications			0.611
Bradypnea	3 (13%)	1 (5%)	
Laryngospasm	0	0	
Desaturation	0	0	
In the post-anesthesia care unit			
Sedation score 1/2/3/4/5/6	2/21/0/1/0/0	0/17/4/0/0/0	0.028
Nausea 1/2/3/4	20/2/0/2	18/3/0/0	0.482
Vomiting	1 (4%)	0	>0.999
Pain (0–10)	4.7 ± 2.2	5.2 ± 1.6	0.367
atients receiving antiemetics	2 (8%)	0	0.491
Patients receiving analgesics	13 (54%)	16 (76%)	0.124

Values are expressed as the mean ± standard deviation or number (%). EtSevo, end-tidal concentration of sevoflurane. Sedation score was recorded by Ramsay Sedation Scale range, 1–6.

## Data Availability

The datasets used and analyzed during the current study are available from the corresponding author upon reasonable request.
